# The role of facilities management in fighting COVID-19 outbreak: Evidence from Malaysian public hospitals

**DOI:** 10.3389/fpsyg.2022.1045972

**Published:** 2023-01-17

**Authors:** Waqas Mehmood, Muhammad Fareed, Rasidah Mohd-Rashid, Muhammad Umair Ashraf, Attia Aman-Ullah

**Affiliations:** ^1^School of Economics, Finance and Banking, Universiti Utara Malaysia, Sintok, Malaysia; ^2^School of Business Management, Universiti Utara Malaysia, Sintok, Malaysia; ^3^Department of Sociology, Government College Women University, Sialkot, Pakistan

**Keywords:** Malaysia, COVID-19 pandemic, healthcare, facilities management interventions, public hospitals

## Abstract

The aim of this study is to provide a holistic review of the fight against COVID-19 in developing countries, particularly Malaysia. Specifically, the study aims to determine how facilities management delivery in public hospitals can be improved to ensure readiness in handling COVID-19 cases. We conducted a review of the literature and reliable media updates on COVID-19 and services management. A critical synthesis of COVID-19 information was conducted to scrutinise the technical aspects and highlight how facilities management can be improved to ensure hospital readiness in managing COVID-19 cases. The data and information used in the present study were collected up to the time of writing this paper, which leaves a room for further studies. Nonetheless, this study’s recommendations are useful for understanding the present and future pandemics. This study is a first attempt to summarise the data on facilities management in relation to the COVID-19 pandemic in the Malaysian context. The study’s findings are suitable for the developing countries in managing healthcare management practices in the fight against COVID-19. This study aims to highlight current issues in order to provide a more objective assessment of facilities management to ensure hospital readiness in handling COVID-19 cases.

## Introduction

**Figure 1:**
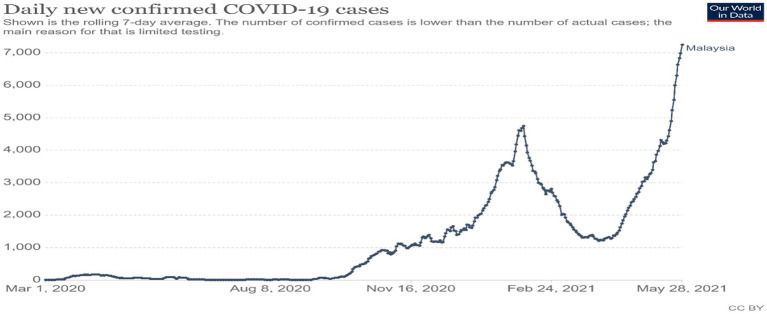
Reproduced under CC-BY-4.0. Our World in Data, COVID-19 Data Explorer - Our World in Data.

**Figure 2:**
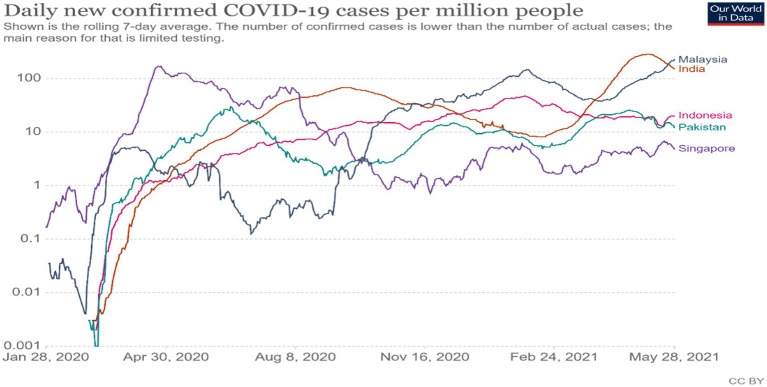
Reproduced under CC-BY-4.0. Our World in Data, Data Explorer - Our World in Data.

The novel coronavirus pandemic took the world by surprise and spread rapidly throughout the world in the end of 2019 and early 2020. This novel coronavirus is known as “SARS-CoV-2” and officially named COVID-19. Initially, a few COVID-19 cases were reported in Wuhan, China. The mass scale of its spread has led the World Health Organization (WHO) to declare it as a global pandemic. To date, about 164 million people have been infected, causing 3.4 million deaths in 216 countries. Governments across the world are making efforts to handle the COVID-19 pandemic to save and treat their populations from the virus while at the same time safeguarding their economies (Goniewicz et al., 2020). The pandemic severely hit many countries in different parts of the world, particularly Italy, Spain, the United Kingdom (UK), the United States of America (USA), and Brazil. The Asian region, including Malaysia, is at the top of COVID-19 statistics with 47,204,344 cases (refer to Figures 1, 2).

Malaysia has recorded a total of 558,534 COVID-19 cases and roughly 8,000 new cases per day since 30 May 2021, with a death toll of more than 2,650 cases. The Star News (2021) reported that millions of lives are at risk due to delays and a lack of access to COVID-19 diagnosis tests, self-protective equipment, and vaccinations. Most of the vaccine supplies have been procured by developed countries, with rich countries receiving more than 87% of the total available vaccines of 700 million doses and developing nations receiving only 0.2% as of mid-April 2021. One-third of the population of the former have been vaccinated in contrast to 1 in 500 of the latter. By mid-May 2021, less than 12 percent of the world population have been vaccinated with ten rich countries receiving 4 out of 5 vaccines. The vaccines produced by Pfizer are mostly supplied to the world’s rich countries despite the pledge by the company’s CEO Alberto Bourla to those poorer countries will have the same access as the rest of the world. WHO statistics have confirmed that Pfizer has done very little for poor countries. By May 2021, patients assessed as having low chances of survival were no longer transferred to intensive care units (ICUs) throughout Malaysia, according to Kedah State Health and Local Government Committee Chairman Datuk Dr. Mohd Hayati Othman. The COVID-19 pandemic has put immense pressure on Malaysia’s healthcare system (Robbins et al., 2020).

By 31^st^ May 2021, more than one million COVID-19 cases had been recorded and Malaysia became the country with the highest number of cases in Asia even surpassing India (Our World in Data, 2021). These statistics indicate the possibility of recording even higher numbers of daily cases in the country in the future if effective actions are not taken. Scholars and experts have alerted the need to vaccinate every eligible individual to curb the spread of the virus. They have also raised various concerns regarding Malaysia’s readiness in fighting the pandemic, painting a gloomy picture of Malaysia’s healthcare system. Malaysia as a developing country experiences a shortage of healthcare practitioners, faces consistent pressure on the present infrastructure and material due to increased utilisation over the years, has low application of information and communications technology (ICT), and faces the issues of low service quality and poor motivation among healthcare workers (Shah et al., 2020; Amir et al., 2021; Hamzah et al., 2021; Hashim et al., 2021). Many of the country’s doctors have moved to developed countries after completing their formal education. Malaysia’s healthcare budget is still below international standards. *Per capita* income is low, restricting the people’s ability to comply with international precautionary measures such as adhering to a complete lockdown, purchasing costly medicines, and wearing face masks.

**Figure 3:**
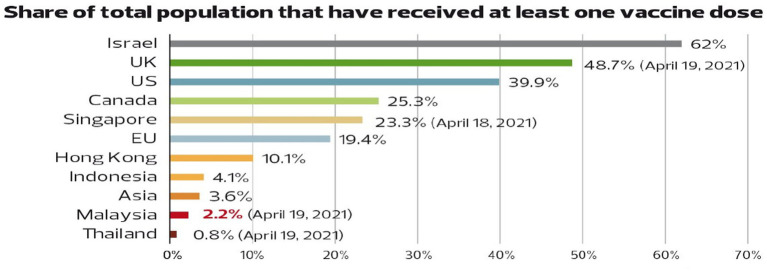
Share of the total population that have received at least one vaccine dose. Reproduced under CC-BY-4.0. Our World in Data, Share of people who received at least one dose of COVID-19 vaccine (ourworldindata.org).

The Edge Market (2021) reported that the Malaysian government launched the national COVID-19 immunisation programme on 24 February 2021. The government used multiple platforms such as the radio, television, and social media to create awareness and encourage Malaysians to register for vaccinations. On the first day of the campaign, more than half of the country’s population registered for vaccination *via* a dedicated app for vaccination known as MySejahtera. By 19 April, about 9.05 million Malaysians had registered for vaccination but only a few had received notifications for vaccination injections. According to Our World in Data, 451,237 people were completely vaccinated as of 19 April, and most of them were frontliners. Another 275,174 people had been administered with one dose, giving a total of about 1.2 million doses administered (the Pfizer vaccine requires two doses per person for full vaccination). In comparison, 23% of the population of the neighbouring country, Singapore, had received at least one dose, which is substantially higher than the 2 and 4% recorded by Malaysia and Indonesia, respectively. The rates are much higher for developed countries such as the US (40%), the UK (49%), Canada (25%), and European Union (19%), while the average for Asia is only 3.6% (refer to Figure 3). According to experts and officials, Malaysia’s progress in handling the COVID-19 pandemic as evidenced by the high rates of severe stage patients and deaths, vaccination uncertainty, and distrust in governmental bodies has caused uncertainty regarding the potential success of internationally funded public health programmes in the country.

The high infection rates and low vaccination rates have led to concerns regarding hospitals’ readiness to handle COVID-19 cases given the present overutilisation of the healthcare system. In the viewpoint of hospital facilities management, the basic function of it is to support and add up the value to the hospital’s main activity of executing medical and clinical diagnosis. For the National Health Service (NHS) of UK, healthcare facility management provides satisfying attention and various soft services that provide for the primary clinical operations of hospitals (NHS, 1998). Healthcare surroundings affect the quality of care provided, patient satisfaction, and sometimes the time taken to recovery. Hospital admirations should ensure continued compliance and consistent preparedness in maintaining a properly operated physical environment that supports the main healthcare services (Lucas et al., 2013). Unstable economic performances and poor healthcare mechanisms in most countries have led to inadequate investments in healthcare systems. This situation has caused concerns regarding the readiness of the healthcare setting in most developing countries in handling this pandemic. Since COVID-19 is a highly transmissible disease, it brings to light the importance of facilities management services in hospitals during the prevalence of the disease (Pinheiro and Luís, 2020). Non-clinical hygiene is related to facilities management practices in waste management, cleaning, and maintenance (Njuangang et al., 2016). Deterioration in facilities management services can cause virus transmission in hospitals through environmentally mediated ways (Dietz et al., 2020). For example, all those individuals using hospital services including visitors, patients, and other users can be exposed to the virus as a result of close-contact with abiotic surface (Rothan and Byrareddy, 2020). The healthcare system is a key component in fighting against COVID-19. However, environmentally mediated pathways for COVID-19 transmission put a danger to hospitals worldwide. This situation is even more relevant in emerging countries such as Malaysia where the hospitals are congested, healthcare facilities are unsanitary, and staff are in severe shortages (Amir et al., 2021; Hashim et al., 2021). These factors increase the likelihood of COVID-19 spreading in healthcare facilities in developing countries. In addition to the non-healthcare mechanisms implemented by the government such as social distancing and closure of areas with high occupancies like schools and workplaces, the authorities should also consider the aspects of facilities management to counter infections in hospitals. Hospital facilities management service has a significant role in establishing public health procedures and lowering the risk of infection. Most of the studies on facilities management interventions do not add much value to the intervention decision-making process for policymakers and managers. Although many studies considered multiple interventions, such as ventilation and occupant density controls, a systematic analysis of interventions’ combined effects is required. Additionally, the cost-effectiveness of various interventions and combinations was not thoroughly investigated in the previous literature (Zhang et al., 2022). Motivated by these issues, this paper offers a conceptual review of how facilities management services can be improved in Malaysian hospitals to protect healthcare workers and manage the COVID-19 pandemic with the assistance from WHO, which is helping by providing equipment for the diagnosis of COVID-19 cases. The Malaysia government has also established a relief fund to collect donations for the welfare of the public as Malaysians place considerable value on human relationships, concern for others, support, and the quality of life (Noor et al., 2018). Henceforth, the outline of extent to better facilities management practices are helpful to reduce risk of infection from COVID-19. This paper contributes to the literature and policymakers by providing a comprehensive review of the interventions and readiness to handle the COVID-19 pandemic by focusing on a highly affected country, i.e., Malaysia. The study also outlines practical measures to improve the facilities management services provision in Malaysian public hospitals. Improving the role of facilities management will enhance the quality and effectiveness of services, which are central towards creating n virus free environment and antivirus established milieu to counter the present pandemic and prevent future virus outbreaks (Megahed and Ghoneim, 2020).

This research is important in a number of ways. Firstly, it gives a perspective of the comprehensive review of the COVID-19 fight in the developing country like Malaysia. Second, this research seeks to investigate how facilities management delivery in public hospitals may be enhanced to assure efficiency in dealing with COVID-19 cases, since facilities management is becoming more important. Third, this research provides ways to improve the performance of facilities management in the healthcare industry.

In addition, this research differs from the previous literature in several ways. First, the comprehensive review related to the fight against COVID-19 offered by this research is noteworthy since it has not been investigated in earlier studies. Second, facilities management is a prevalent concern in the health-care industry since hospital facilities management services play an important part in developing public health procedures and minimising infection risk. However, most research on facility management interventions do not contribute much value to policymakers’ and managers’ intervention decision-making processes. Furthermore, the prior research did not extensively evaluate the cost-effectiveness of facilitation management interventions (Zhang et al., 2022). Third, this research contributes to the literature on facility management in Malaysia.

## Materials and methods

Due to the risk associated with collecting primary data from hospitals, this study presents a vital review of the prominent literature on facilities management and COVID-19 based on credible news and previous studies on facilities management in Malaysian public hospitals. We reviewed and analysed the previous studies on COVID-19, especially those that addressed the issues of virus free environment. Additionally, different government addresses and reports as well as reliable organisational updates and news feeds from WHO and the Ministry of Health Malaysia were used. The aim of this research is to capture the interventions and experiences as they happened. However, since experiences change quickly over time, some statements and interventions reviewed in this paper are case driven.

## Previous literature on healthcare facilities management and COVID-19

The previous literature is reviewed in two different segments. The first section provides a rapid review of healthcare facilities management, and the second section inspects Malaysia’s responses and readiness to counter the COVID-19 pandemic. The first section is important to inform readers on healthcare facilities management as it establishes the extensive and theoretical interests of the study. The second section is geared towards assessing the present interventions to determine whether the relevant authorities pay attention to facilities management. This is to meet the first goal of the study, following which proper interventions to develop facilities management delivery are then drawn.

### Brief review of healthcare facilities management

The NHS describes healthcare facilities’ management as ‘the process by which NHS trust creates and sustains a caring environment and delivers quality services to meet clinical needs at best cost’ (NHS, 1998). Healthcare facilities management is considered one of the most multifaceted and challenging tasks to accomplish due to the need to provide efficient service every time. The slightest error may lead to complications, even resulting in death. Generally, hospital facilities management services encompass a combination of both soft and hard services, where soft services management deals with waste management, cleaning, landscaping, and safety. Rodríguez-Labajos et al. (2018) acknowledged the increasing importance of facilities management and other aspects of healthcare administrations. Facilities management impacts the quality and efficiency of healthcare services by being responsible for maintaining hospital structure and services. Facilities management has also become a significant aspect of healthcare management due to innovations in healthcare, development of facilities management, expenditures and budgetary hurdles, and epidemiological problems that require hospital infrastructures and services to react to such situations (Shohet and Nobili, 2017). Resources complexities, such as treating facilities management and properties as cost centres, have led to neglecting this aspect of healthcare provision in many parts of the developing world (Amos et al., 2020). Currently, facilities management faces numerous issues. Among them are irresponsive facilities management practices, unstable policy and strategies, non-existing building maintenance manuals, insufficient funding, a lack of guidance, and socio-cultural barriers (Koleoso et al., 2017). Such issues have led to healthcare remedial environment with inefficient facilities, resulting in hospital-acquired infections (Amankwah et al., 2019). Hospital performances are also reported in terms of the ability to handle problems during the time of infectious disease spread as they have the responsibility to treat and save their patients. The difficulty to practise social distancing measures in the healthcare setting under the current circumstances underlines the importance of facilities management provision.

### The role of facilities management in strategies to combat COVID-19

This section attempts to provide an overview of the readiness to handle the COVID-19 pandemic with a special focus on the Malaysian population. WHO has reported that the COVID-19 pandemic is a serious danger to the social dynamics, financial growth, and safety of Malaysia (World Health Organization, 2020). COVID-19, which first appeared in China, reached Malaysia within 2 months and caused panic to the people. Initially, Asian countries handled the pandemic relatively well but due to weak management, inadequate resources, and a lack of awareness, the countries’ situation worsened over time. Since April 2021, many countries have banned the entry of travellers from high-risk countries, and Malaysia is also included in the high-risk countries list. In the Asian region, the new COVID-19 cases reported by Malaysia are close to those reported by India, Pakistan, and Bangladesh. Moreover, Malaysia has comparatively low testing capabilities and experiences delays in detecting positive COVID-19 cases. The Malaysian government’s strategic response to manage COVID-19 is focused on:

- Consolidating regional coordination with global and national organisations.
- Establishing membership capability.
- Strengthening public knowledge by way of communication.
- Accelerating research and development for doctor-patient, vaccine development, and diagnosis.
- Continuous monitoring and forecasting (WHO, 2020).

The Malaysian government handles the pandemic by focusing on six areas: diagnosis in laboratories, observation involving screening at the point of entrance and cross-border activities, prevention of infection and quarantine in healthcare facilities, medical treatment of people with severe COVID-19 symptoms, communication, and supply chain management. On 23 April 2020, WHO, as a worldwide online event was arranged; where it is able to increase USD595 million to counter the spread of COVID-19.

Malaysia’s readiness has improved with the funding from WHO amounting to USD595 million, which is used to increase testing capabilities, support the training of medical staff, establish labs, and purchase personal protection equipments (PPEs). The International Monetary Fund (IMF) has also provided financial assistance of USD1.4 billion to Malaysia to assist with building capacities for the fight against COVID-19. Moreover, several other countries such as China and Russia and organisations such as Pfizer and Oxford-AstraZeneca have contributed vaccines to Malaysia.

In the country’s effort to control the spread of the pandemic, Malaysia has taken some drastic actions including closing schools, forbidding eating out as restaurants and social gatherings, shutting down markets, allowing only specific types of shops to open, i.e., medical, food, and essentials, and limiting the number of people per vehicle. Despite these measures, the numbers of new and active cases continue to rise over time, putting extensive pressure on the country’s healthcare system.

The healthcare sector in most Asian countries including Malaysia was overstressed even before the pandemic. The healthcare industry in Malaysia contributes 10% to the gross domestic product (GDP; Shahzad et al., 2020). There is no proper allocation for healthcare expenses, and these countries often allocate relatively small budgets on healthcare. Besides, health insurance is not popular in this region, which means that most people are paying for healthcare expenditures from their pockets. For example, in Malaysia, 65% of the healthcare expenditures are paid out of pocket due to unstable institutional healthcare assurance. The Malaysia healthcare system is also experiencing serious issues such as staff shortage, poor healthcare settings, low financial budgets, low *per capita* income, and poor law and order with weak institutions. Consequently, Malaysia faces problems in treating COVID-19 patients. Many Malaysians cannot afford to buy face masks or adopt other precautionary measures. Hence, COVID-19 cases continue rise and overload the country’s healthcare system. Additionally, regarding to the high number of cases and deaths, the pandemic has imposed unprecedented pressure on the present healthcare structure. As a result, temporary quarantine centres have been established, with numerous empty buildings, public centres, and schools transformed into quarantine centres in Malaysia and other Asian countries. Noticeably, the healthcare structure in Asian countries, particularly Malaysia, is still not ready to deal with the pandemic. There seems to be inadequate effort and information on healthcare facilities management. Current intervention measures seem to focus on financial and non-healthcare mechanisms such as social distancing. While monetary and social interventions are important to assist the vulnerable groups, it is imperative to establish an anti-free-virus healthcare environment due to hospitals’ core responsibility to treat and care for patients. Furthermore, there needs to be a balance between social, economic, and healthcare facilities management aspects to generate a robust healthcare system that can deal with the present and future pandemics.

## Way forward

The COVID-19 pandemic and other potentially new outbreaks require a holistic perspective in order to find feasible solutions. Undoubtedly, one of the most fundamental changes is in healthcare environment. Given the persistent increase in infection cases that may further pressure the already strained healthcare settings, is necessary to introduce interventions to improve the facilities management performance in the long term. This paper provides some recommendations for these interventions.

### Lower physical proximity of the staff of facilities management through work scheduling

The major concern currently is the appointment and readiness of the hospital staff, comprising both medical and non-medical facilities staff, to show up at work and expend the effort (Goniewicz et al., 2020). The coronavirus is infectious and easily transmissible, and awareness about how the virus spreads is still emerging. As per previous researchers, every tangible surface in hospitals that treat COVID-19 patients are contaminated (Sizun et al., 2000). Additionally Ong et al. (2020) provided evidence of rooms where COVID-19 patients were quarantined showing widespread environmental contamination. Therefore, hospital staff face a high risk of getting infected by the virus on site or when executing their tasks. Also, it has been found that close contact during meetings is a major source of virus transfer (Dietz et al., 2020).

### Provide intensive training and resources

Healthcare facilities workers work in a different surrounding that is comparatively compound and multidimensional, requiring critical thinking, focus, and efficiency. Hence, the government and hospital administration need to deliver frequent and up-to-date training on new approaches for virus control and the expected outcomes. Asset management such as the safety of equipment should be prioritised by the facilities management service team. Adequate training and resources will improve the performance of facilities management staff and have positive impacts on frontline healthcare workers, which will reduce the prevalence of infections in hospitals. Organizations typically have limited incentives to capitalize in distinct pandemic management facilities since there is less likelihood of such events. And although firms are likely to refresh resilience plans in combating pandemic, it is important to ponder changes in today’s environment.

### Change management

The COVID-19 pandemic provides the opportunities for facilities management to practise change management which is crucial for organisational performance. Pinheiro and Luís (2020) stated that healthcare facilities management should be capable of adopting changes to cater to the COVID-19 operational requirements, and changes must be made to other domains that are affected by the pandemic. Changes to the work setting must be in the multi-facet style and should incorporate environmental, social, and financial features of virus management with the objective of reducing the probability of virus transmission. Hence, the facilities management department need to implement the required changes swiftly to incorporate the new mechanisms of service delivery. For example, the pandemic has necessitated the introduction of new work norms such as refilling hand sanitizer, performing surface sanitisation on a regular basis, and using face masks. Housekeeping staff in charge of waste management and cleaning need to be re-orientated to enable them to adapt to these new shifts.

### Stakeholder communication

In the past years, communication gap has been observed between the government’s strategic planning and facilities management operations. Real-time information should be provided promptly in response to any queries. Likewise, healthcare facilities processes should be related with the facilities management to recognize interactions and commonalities that might have life-threatening consequences for the efficient running of healthcare delivery and patient safety (Lucas et al., 2013). An appreciative association between facilities management of healthcare delivery will enable the healthcare team to improve the quality and speed of decision-making, thus preventing costly interruptions to the healthcare delivery procedure. Established protocols and guidance on the pandemic should be communicated to facilities management staff on a daily basis. Given that the way to death COVID-19 is presently not recognized and assumed that any future strategy would be dependent upon technical predicting, fast, clear and relevant information on the success established and required interventions must be properly communicated to all hospital stakeholders. Also, the facilities management team need to design a methodical approach for collaboration with wider stakeholders. Facilities management sub stakeholders, e.g., subcontractors must be informed of any changes to the preferred service delivery. Poor ICT infrastructure is a challenge for many developing countries given the important role of technology in improving communication. In the absence WhatsApp chats, dedicated hotlines and daily meetings should be promoted.

### Build a key coordination channel to improve strategic facilities management

According to Amos et al. (2020), there is a need to improve facilities management for better hospital performance. In the current situation, it is crucial for public hospitals to enhance the strategic role of their facilities management department. Therefore, there is a need to build a for facilities management activities. The broad functions of facilities management require many sub-unit heads within hospitals. In many healthcare institutions, health service administrations are by default made up of the general heads of the non-clinical facility. Some healthcare administration personnel might not be well versed in facilities management, does not encouraging strategic role with an argumentative problem in post governmental hospitals in emerging countries. The recommended coordination is intended to serve the strategic interest of facilities management by coordinating the different facilities management components. Undoubtedly, a lack of central cooperation has led to inefficiencies in executing the critical functions of facilities management. With a promoted strategic role in healthcare management, facilities management will be able to articulate clear strategies in order to achieve the objectives and realise the shared values of hospitals, with the overall aim of improving hospital performance.

### Establish a facilities management database for space management

Public administered hospitals need to establish a database on the healthcare environment, as it has been observed that most of the hospitals in emerging countries do not have data on their healthcare facilities. An updated database will enable hospitals to identify areas of space over-utilisation. Numerous scientific studies have postulated that the coronavirus transmits easily in crowded areas. Highly used areas in hospitals often become a potential ground for virus transmission due to frequent contacts, especially in shared spaces and on surface for instance washrooms and door handles (Horve et al., 2020). Given the increasing need to isolate patients in hospitals, space management has become a major issue in hospital facilities management. Hence, a database showing the total space in the hospital and space per healthcare provision is needed. Highly reliable data on facilities will benefit resources management and can help with facility planning and activity preparation for example replenishment of sanitizers, general cleansing and disinfection plans, and proper cleaning. Furthermore, it will provide valuable input in facilities strategic planning, e.g., design briefings and makeovers.

### Improve the standards of cleaning and daily decontamination

Ong et al. (2020) stated that the coronavirus can remain on surface till 9 days at room temperature and even up to weeks on surfaces with lower temperatures. Hence, thorough and frequent cleaning is required to disinfect the surfaces, particularly for surfaces in public areas where there are higher contacts (Eykelbosh, 2020). Additionally, facilities management should ensure adequate supplies of necessary hygiene items for instance running water and hand sanitizers and provide clear instructions using signage, automated screens, and other resources.

### An approach towards a post-pandemic healthcare setting

It has been argued that whether the coronavirus will continue to exist for an indefinite period is partially dependent on mass vaccinations or treatments of COVID-19. While there are prominent consequences in the initial phases of vaccine development and diagnosis, it is important for the healthcare system such as facility management to consider the post-pandemic healthcare settings. Megahed and Ghoneim (2020) proposed that the “new normal” will affect the planning and designing of the healthcare environment and have also suggested that safety environment in the advanced healthcare system must be enhanced to prevent infections. Therefore, healthcare facility personnel need to establish a strategic team that will plan upcoming projects with a resilient viruse-free environment. In line with this, (Kashdan, 2020) illustrated the use of disinfected and uncontaminated material that can be used effortlessly to sanitise, while Molla (2020) advocated that the focus should be on modern skills that can maintain consistently high space temperatures to eliminate and kill harmful germs and viruses. Currently, Malaysia is highly dependent on imported medicines, which increase the prices of medicines for treating COVID-19 patients. Hence, Malaysia needs to encourage local manufacturers to produce medicines and should allocate budgets for research and development in this area. In sum, multi-dimensional collaborations are needed to develop ground-breaking solutions for the post-pandemic healthcare setting.

### Establishing crisis management team

The top priority for the businesses and government authorities during a crisis is the well-being of the people. Businesses can contribute and complement these efforts by having a professional crisis management team. A strong crisis management team can help businesses to ensure the well-being of their people, keep the resources protected and reinforce their ability to perform optimally when the odds are stacked against them. From a perspective of risk management, volatile nature of epidemics makes crisis management worthless in preventing its implications. However, with the proper financial and human resources in place and with the right skills we can built a more resilient environment where we can combat any future crisis. Coming out of this pandemic, organizations have learnt to be resilient and prepared. Yet a more balanced approach requires to be held between economic, social, and health-care facilities management to build a resilient healthcare environment to cope with the existing pandemic as well as any such future crisis. Organizations must prepare crisis management agency and response, involving C-suite executives and delegations of authorities, so that delegations are well planned to implement timely decisions in the setting where key decision-makers are not available.

## Conclusion

Malaysia was one of the highly affected countries by the COVID-19 and yet COVID-19 cases are surging once again. The overstrained and inadequate healthcare infrastructure along with poor facilities management practices underlines the necessity to put forward a technical view on how to handle the pandemic in the healthcare setting. This review shows that present interventions are largely on non-healthcare mechanisms such as social distancing, while facilities management interventions have been lacking despite its critical role in managing COVID-19 cases in public hospitals. Even though there are many published studies on coronavirus, very few scientific studies have focused on COVID-19 and facilities management. Most of the previous studies focused on medicine, treatment, vaccine, public health, and the related factors. The paper adds to the literature by explaining the interventions and receptiveness to the COVID-19 pandemic in Asia. It also argues for including facilities management in the present interventions to counter the pandemic. This study on the virus advances alongside present socio-economic methods, an irrepressible healthcare structure must be established due to the responsibility of healthcare facilities to treat patients. Healthcare stakeholders in developing countries are advised to incorporate the important endorsements facilitation management strategies to improve healthcare structure.

## Future research directions

Despite the best efforts from the authors, this research has limitations, including the likelihood that the research might have overlooked a variety of related publications. Nevertheless, this paper serves as the basis for future research on facilities management solutions for battling the pandemic and improving post-pandemic building operations. More studies on facilities management interventions, receptiveness to the COVID-19 pandemic are required for future respiratory infection control. Malaysia as a developing country with limited resources faces mounting challenges in responding to the crisis management. Future researches can also be carried out to explore the rapid strategies in dealing with such challenges to comprehend crisis in developing countries.

## Public interest statement

The COVID-19 pandemic has provided unexceptional encounters and authenticities to the healthcare system in Malaysia. While governments in the Malaysia have made substantial efforts to curb the spread of the pandemic, there is still a need to develop a strategic services management plan to improve hospital management of COVID-19 cases. A facilities management system that is timely, effective, and legally sound is a necessary feature for virus-free environment. The COVID-19 pandemic is an on-going concern, and the situation worldwide continues to change rapidly overtime. This study aims to highlight current issues in order to provide a more objective assessment of facilities management to ensure hospital readiness in handling COVID-19 cases.

## Author contributions

WM: formal analysis. RM-R: investigation. MA: methodology. MF: editing, proof reading, and project administration. AA-U: writing – original draft. All authors contributed to the article and approved the submitted version.

## Conflict of interest

The authors declare that the research was conducted in the absence of any commercial or financial relationships that could be construed as a potential conflict of interest.

The reviewer AK declared a shared affiliation with the authors WM, MF, RM-R, and AA-U to the handling editor at the time of review.

## Publisher’s note

All claims expressed in this article are solely those of the authors and do not necessarily represent those of their affiliated organizations, or those of the publisher, the editors and the reviewers. Any product that may be evaluated in this article, or claim that may be made by its manufacturer, is not guaranteed or endorsed by the publisher.

## References

[ref1] Amankwah O. Weng-Wai C. Mohammed A. H. (2019). Modelling the mediating effect of health care healing environment on core health care delivery and patient satisfaction in Ghana. Environ Health Insights 13:117863021985211. doi: 10.1177/1178630219852115 PMC6560799 31217690

[ref2] Amir A. R. M. N. Nordin A. B. A. Lim Y. C. Shauki N. I. B. A. Ibrahim N. H. B. (2021). Workforce mobilization from the National Institutes of Health for the Ministry of Health Malaysia: a COVID-19 pandemic response. Front. Public Health 9:574135 doi: 10.3389/fpubh.2021.574135 33643985 PMC7905027

[ref3] Amos D. Musa Z. N. Au-Yong C. P. (2020). Performance measurement of facilities management services in Ghana’s public hospitals. Build. Res. Informat. 48, 218–238. doi: 10.1080/09613218.2019.1660607

[ref4] Dietz L. Horve P. Coil D. Fretz M. Eisen J. Van Den Wymelenberg K. (2020). Novel coronavirus (COVID-19) pandemic: built environment considerations to reduce transmission. mSystems 5, e00245–e00220. 32265315 10.1128/mSystems.00245-20 PMC7141890

[ref5] Edge Market (2021). Available at: https://www.theedgemarkets.com/article/special-report-why-malaysia-lagging-behind-vaccination-rollout

[ref6] Eykelbosh A. (2020). COVID-19 Precautions for Multi-Unit Residential Buildings. National Collaborating Centre for Environmental Health: Winnipeg, MB, Canada

[ref7] Goniewicz K. Khorram-Manesh A. Hertelendy A. J. Goniewicz M. Naylor K. Burkle F. M. (2020). Current response and management decisions of the European Union to the COVID-19 outbreak: a review. Sustainability 12:3838. doi: 10.3390/su12093838

[ref8] Hamzah N. M. Yu M. M. See K. F. (2021). Assessing the efficiency of Malaysia health system in COVID-19 prevention and treatment response. Health Care Manag. Sci. 24, 273–285. doi: 10.1007/s10729-020-09539-9 33651316 PMC7921615

[ref9] Hashim J. H. Adman M. A. Hashim Z. Radi M. F. M. Kwan S. C. (2021). COVID-19 epidemic in Malaysia: epidemic progression, challenges, and response. Front. Public Health 9:560592. doi: 10.3389/fpubh.2021.560592 34026696 PMC8138565

[ref10] Horve P. F. Lloyd S. Mhuireach G. A. Dietz L. Fretz M. MacCrone G.. (2020). Building upon current knowledge and techniques of indoor microbiology to construct the next era of theory into microorganisms, health, and the built environment. J. Expo. Sci. Environ. Epidemiol. 30, 219–235. doi: 10.1038/s41370-019-0157-y, PMID: 31308484 PMC7100162

[ref11] Kashdan R. (2020). Six ways urban spaces may change because of coronavirus. Available at: https://www.bostonmagazine.com/property/2020/04/30/urban-spaces-coronavirus/ (Accessed May 8, 2020).

[ref12] Koleoso H. A. Omirin M. M. Adewunmi Y. A. (2017). Performance measurement scale for facilities management service in Lagos-Nigeria. J. Facil. Manag. 15, 128–152. doi: 10.1108/JFM-04-2016-0015

[ref13] Lucas J. Bulbul T. Anumba C. (2013). Gap analysis on the ability of guidelines and standards to support the performance of healthcare facilities. J. Perform. Constr. Facil. 27, 748–755. doi: 10.1061/(ASCE)CF.1943-5509.0000364

[ref14] Megahed N. A. Ghoneim E. M. (2020). Antivirus-built environment: lessons learned from Covid-19 pandemic. Sustain. Cities Soc. 61:102350. doi: 10.1016/j.scs.2020.102350, PMID: 32834930 PMC7313520

[ref15] Molla R. (2020). This is the end of the office as we know it. *Vox*, 14.

[ref16] NHS E. (1998). Health Facilities Note (HFN) 17—A Business Approach to Facilities Management. London: HMSO Publications.

[ref17] Njuangang S. Liyanage C. L. Akintoye A. (2016). Performance measurement tool (PMT) to control maintenance-associated infections. Facilities 34, 766–787. doi: 10.1108/F-12-2014-0107

[ref18] Noor W. S. W. M. Fareed M. Isa M. F. M. (2018). Examining cultural orientation and reward management practices in Malaysian private organizations. Pol. J. Manag. Stud. 18, 218–240. doi: 10.17512/pjms.2018.18.1.17

[ref19] Ong S. W. X. Tan Y. K. Chia P. Y. Lee T. H. Ng O. T. Wong M. S. Y.. (2020). Air, surface environmental, and personal protective equipment contamination by severe acute respiratory syndrome coronavirus 2 (SARS-CoV-2) from a symptomatic patient. JAMA 323, 1610–1612. doi: 10.1001/jama.2020.3227, PMID: 32129805 PMC7057172

[ref600] Our World in Data (2021). Available at: https://ourworldindata.org/coronavirus#explore-the-global-situation

[ref20] Pinheiro M. D. Luís N. C. (2020). COVID-19 could leverage a sustainable built environment. Sustainability 12:5863. doi: 10.3390/su12145863

[ref21] Rodríguez-Labajos L. Thomson C. O’Brien G. (2018). Performance measurement for the strategic management of health-care estates. J. Facil. Manag. 16, 217–232. doi: 10.1108/JFM-10-2017-0052

[ref700] Robbins T. Hudson S. Ray P. Sankar S. Patel K. Randeva H.. (2020). COVID-19: A new digital dawn? Digit. Health 6, 1–3. doi: 10.1177/2055207620920083, PMID: 32313668 PMC7153182

[ref22] Rothan H. A. Byrareddy S. N. (2020). The epidemiology and pathogenesis of coronavirus disease (COVID-19) outbreak. J. Autoimmun. 109:102433. doi: 10.1016/j.jaut.2020.102433, PMID: 32113704 PMC7127067

[ref23] Shah A. U. M. Safri S. N. A. Thevadas R. Noordin N. K. Abd Rahman A. Sekawi Z.. (2020). COVID-19 outbreak in Malaysia: actions taken by the Malaysian government. Int. J. Infect. Dis. 97, 108–116. doi: 10.1016/j.ijid.2020.05.093, PMID: 32497808 PMC7264933

[ref24] Shahzad A. Hassan R. Abdullah N. I. Hussain A. Fareed M. (2020). COVID-19 impact on e-commerce usage: an empirical evidence from Malaysian healthcare industry. Hum. Soc. Sci. Rev. 8, 599–609. doi: 10.18510/hssr.2020.8364

[ref25] Shohet I. M. Nobili L. (2017). Application of key performance indicators for maintenance management of clinics facilities. Int. J. Strateg. Prop. Manag. 21, 58–71. doi: 10.3846/1648715X.2016.1245684

[ref26] Sizun J. Yu M. Talbot P. (2000). Survival of human coronaviruses 229E and OC43 in suspension and after drying onsurfaces: a possible source of hospital-acquired infections. J. Hosp. Infect. 46, 55–60. doi: 10.1053/jhin.2000.0795, PMID: 11023724 PMC7134510

[ref27] Star News (2021). Available at: https://www.thestar.com.my/aseanplus/aseanplus-news/2021/05/20/prof-jomo-millions-to-die-from-vaccine-deprivation-as-company-profits-come-before-people

[ref800] World Health Organization (2020). Available at: https://www.who.int/publications/m/item/malaysia-strong-preparedness-and-leadership-for-a-successful-covid-19-response

[ref29] Zhang Y. Hui F. K. P. Duffield C. Saeed A. (2022). A review of facilities management interventions to mitigate respiratory infections in existing buildings. Build. Environ. 221:109347. doi: 10.1016/j.buildenv.2022.109347, PMID: 35782231 PMC9238148

